# In vitro analysis of the sealing ability of different root canal sealers under thermo-mechanical stress

**DOI:** 10.6026/973206300211518

**Published:** 2025-06-30

**Authors:** Antriksh Azad, Vaishali Mashalkar, Ruchi Agrawal, Yihan Fu, Dipak Chaudhari, Abhishek Haldia, Miral Mehta

**Affiliations:** 1 Department of Conservative Dentistry and Endodontics, College of Dental sciences and Hospital, Rau, Indore, Madhya Pradesh, India; 2 Department of Periodontology, School of Dental Sciences, Krishna VishwaVidyapeeth (Deemed to be University),Taluka-Karad, Dist-Satara - 415 539, Maharashtra, India; 3 Department of Microbiology, Bundelkhand medical College Sagar, Madhya Pradesh, India; 4 Department of General Dent, Smilebuilderz, Lancaster, Pennsylvania, United States; 5 Department of Pediatric and Preventive Dentistry, RUHS College of Dental Sciences, Jaipur, Rajasthan, India; 6 Department of Pediatric and Preventive Dentistry, Karnavati School of Dentistry, Karnavati University, Gandhinagar, Gujarat, India

**Keywords:** Root canal sealers, bioceramic, microleakage, thermomechanical stress, in vitro, apical sealing

## Abstract

The ability to create a proper seal by testing three root canal sealers under conditions that replicated thermal and mechanical stresses is of interest. A total of sixty human single-rooted teeth received epoxy resin-based and calcium silicate-based and bioceramic-based sealers before thermocycling and mechanical loading procedures. Microleakage examinations demonstrated that bioceramic sealer allowed the least dye penetration at 0.45 ± 0.12 mm while calcium silicate sealer permitted 0.71 ± 0.18 mm penetration and epoxy resin sealer ended with 1.02 ± 0.21 mm penetration. The statistical analysis using a One-way ANOVA method established significant differences at a p value less than 0.05. The use of bioceramic sealers leads to high-quality apical barriers which enhances continued endodontic treatment success.

## Background:

The successful outcome of root canal therapy depends on performing complete cleaning and shaping while achieving complete three-dimensional obturation of the root canal system to stop infection and allow healing [1]. The main objective of obturation involves establishing an air-tight barrier which stops microorganisms along with their metabolic waste from entering periapical tissues [2]. The structure of root canal materials suffers challenges because of the dynamic conditions in the oral cavity which includes temperature changes and mechanical forces that come from chewing [3]. Only root canal sealers effectively maintain a tight bond between core material and root canal dentinal walls. Manufacturers have produced different root canal sealer types starting with epoxy resin-based materials followed by calcium silicate-based and finally bioceramic-based materials. The wide popularity of epoxy resin-based sealants stems from their reliable bond quality and minimal dissolution but shrinkage creates potential damage to the root canal seal [4]. The combination of biocompatibility and dimensional stability among bioceramic along with calcium silicate-based sealers enables chemical attachment to dentin [5, 6]. Subjecting root canal filling materials to thermomechanical stress that simulates the changes between oral temperatures and chewing forces affects the physical characteristics and sealing properties [7]. The examination of different sealers under simulated stress exposure helps establish their practical efficiency as well as their expected longevity. The field of root canal sealants has evolved toward dual enhancement of sealant effectiveness alongside their adaptive connection with dentinal components. Strong bonds between materials along with proper adaptation at the interface serve as essential characteristics to prevent material displacement and leakage during the long-term period. Bioceramic-based sealers show encouraging properties that allow them to set up while absorbing moisture while simultaneously releasing biologically active ions which promote tissue restoration and new mineral development [8, 9]. Bioactivity levels between calcium silicate-based sealers and their potential high solubility affect their prolonged sealing capability according to research [10]. The clinical durability of resin-based sealers remains at risk because exposure to thermal stress and time-dependent polymer degradation [11]. Laboratory simulations must evaluate thermal-mechanical stress on obturated root canals because eating hot or cold foods combined with masticatory forces cause interface sealer-dentin expansion and contraction and micro-crack development [12, 13]. Therefore, it is of interest to report the in vitro analysis of the sealing ability of different root canal sealers under thermo-mechanical stress.

## Materials and Methods:

## Sample selection and preparation:

Sixty freshly extracted human single-rooted permanent teeth with mature apices and straight canals were selected. Teeth with cracks, caries, or resorptive defects were excluded. The teeth were decoronated using a diamond disc under water cooling to obtain standardized root lengths of 15 mm. Working lengths was established by inserting#10 K-file into the canal until visible at the apex and subtracting 1 mm.

## Canal preparation:

Root canals were instrumented using a rotary nickel-titanium file system (ProTaper Universal, Dentsply Maillefer, Switzerland) up to size F3. Irrigation was performed with 2.5% sodium hypochlorite between each file, followed by 17% EDTA for smear layer removal and a final rinse with distilled water. All canals were dried with paper points.

## Group allocation and obturation:

Specimens were randomly divided into three groups (n=20) based on the sealer used:

[1] Group A: Epoxy resin-based sealer (AH Plus, Dentsply)

[2] Group B: Calcium silicate-based sealer (MTA Fillapex, Angelus)

[3] Group C: Bioceramic-based sealer (EndoSequence BC Sealer, Brasseler)

Obturation was performed using the lateral condensation technique with gutta-percha cones compatible with the master apical file size. The coronal access was sealed with temporary filling material (Cavit-G).

## Thermo-mechanical stress simulation:

All samples were stored in 100% humidity at 37° for 7 days to allow sealer setting. The specimens were then subjected to thermo-cycling between 5° and 55° for 500 cycles with a dwell time of 30 seconds in each bath. Following thermal cycling, the roots were mounted in acrylic blocks and subjected to mechanical loading using a chewing simulator applying 50 N of vertical force for 100,000 cycles to simulate masticatory forces.

## Dye penetration analysis:

Following stress simulation, the root surfaces were coated with nail varnish, leaving 1 mm around the apex uncoated. The specimens were immersed in 2% methylene blue dye for 48 hours. After rinsing and drying, the roots were sectioned longitudinally, and the extent of dye penetration was measured from the apex using a stereomicroscope at 20x magnification. The mean penetration depth was recorded in millimetres.

## Statistical analysis:

Data were analyzed using SPSS version 26.0 (IBM Corp., Armonk, NY). One-way ANOVA followed by post hoc Tukey's test was used to compare mean dye penetration between groups. A p-value of <0.05 was considered statistically significant.

## Results:

All specimens in the three groups showed varying degrees of dye penetration. The mean values and standard deviations of dye penetration for each group are presented in Table 1 (see PDF). The bio-ceramic-based sealer group (Group C) showed the least mean dye penetration (0.45 ± 0.12 mm), followed by the calcium silicate-based sealer group (Group B) (0.71 ± 0.18 mm), while the highest leakage was observed in the epoxy resin-based sealer group (Group A) (1.02 ± 0.21 mm). Statistical analysis using one-way ANOVA indicated a significant difference in dye penetration among the groups (p < 0.05). Post hoc Tukey's test revealed that Group C had significantly less micro-leakage than Group A (p = 0.001) and Group B (p = 0.042). The difference between Group A and Group B was also statistically significant (p = 0.036) (Table 2 - see PDF).

## Discussion:

A strong apical seal remains essential for root canal treatment success because it halts bacteria along with oral fluids from r eaching periapical tissues [1]. The present laboratory investigation evaluated three popular root canal filling materials including epoxy resin-based and calcium silicate-based and bioceramic-based for their ability to resist thermomechanical changes. Bioceramic-based sealers exhibited reduced microleakage levels to a significant degree than the other tested sealers. Bioceramic sealers exhibit exceptional tightness because they create hydroxyapatite when setting so they establish chemical bonds with dentinal walls [2, 3]. Both properties enable better adaptation of these materials to the canal walls while the filling material [4]. Under difficult conditions like thermal and mechanical stress the mentioned properties help minimize formation of interfacial voids along with microgaps [5]. The high usage of epoxy resin-based sealers is attributed to their excellent flow characteristics and adhesive properties but these positive qualities cause polymerization shrinkage and impaired bonding to moist dentin tissue which resulted in increased dye penetration in Group A [6, 7]. The sealer-dentin interface in resin-based materials tends to degrade during thermocycling because of thermal mismatch between them [8]. The sealer performance of calcium silicate exceeded epoxy resin but both provided less effective results than bioceramic sealers. Research by previous authors indicates that calcium silicate-based materials demonstrate excellent biocompatible and dimensional stable properties although their moisture sensitivity affects their sealing capacity [9, 10]. The reaction process of these materials in the canal depends on the moisture content but this can lead to problems with the apical seal [11]. The study applied thermo-mechanical stress as a method to replicate the conditions found within the oral cavity such as temperature changes and biting forces because these elements threaten the durability of root canal fillings over time [12]. Scientific research shows that a combination of thermocycling and dynamic loading procedures induce material fatigue which might cause marginal leakage [13, 14]. The research team employed dye penetration as the evaluation method for measuring microleakage. The method is accepted by many researchers for laboratory testing but presents challenges because specimen procedures and reading techniques are easily affected [15]. The limitations in this study do not diminish the important findings about material sealing behavior when evaluating groups under simulated clinical settings.

## Conclusion:

Data is in line with recent studies which demonstrate that bioceramic sealers create superior dental chamber seals particularly during exposure to thermal and mechanical factors. Extending the research with long-term aging methods while performing clinical assessments alongside microbial leakage tested is necessary for final validation of these findings.
